# Inhaled nitric oxide and the risk of renal dysfunction in patients with acute respiratory distress syndrome: a propensity-matched cohort study

**DOI:** 10.1186/s13054-016-1566-0

**Published:** 2016-11-30

**Authors:** Sheng-Yuan Ruan, Hon-Yen Wu, Hsien-Ho Lin, Huey-Dong Wu, Chong-Jen Yu, Mei-Shu Lai

**Affiliations:** 1 Institute of Epidemiology and Preventive Medicine, National Taiwan University, No.17 Xu-Zhou Road, Taipei, 10020 Taiwan; 2 Division of Pulmonary and Critical Care Medicine, Department of Internal Medicine, National Taiwan University Hospital, Taipei, Taiwan; 3 Division of Nephrology, Department of Internal Medicine, Far Eastern Memorial Hospital, New Taipei City, Taiwan; 4 School of Medicine, National Yang-Ming University, Taipei, Taiwan

**Keywords:** Acute respiratory distress syndrome, Adverse effect, Nitric oxide, Renal failure, Treatment

## Abstract

**Background:**

Inhaled nitric oxide (iNO) is a rescue therapy for severe hypoxemia in patients with acute respiratory distress syndrome (ARDS). Pooled data from clinical trials have signaled a renal safety warning for iNO therapy, but the significance of these findings in daily clinical practice is unclear. We used primary data to evaluate the risk of iNO-associated renal dysfunction in patients with ARDS.

**Methods:**

We conducted a cohort study using data from a tertiary teaching hospital to evaluate the risk of incident renal replacement therapy (RRT) in iNO users compared with that of non-users. Propensity score matching and competing-risks regression were used for data analysis. Residual confounding was assessed by means of a rule-out approach. We also evaluated effect modification by pre-specified factors using stratified analysis.

**Results:**

We identified 547 patients with ARDS, including 216 iNO users and 331 non-users. At study entry, 313 (57.2%) patients had moderate ARDS and 234 (42.8%) had severe ARDS. The mean patient age was 63 ± 17 years. The crude hazard ratio of the need for RRT in iNO users compared with non-users was 2.23 (95% CI, 1.61–3.09, *p* < 0.001). After propensity score matching, there were 151 iNO users matched to 151 non-users. The adjusted hazard ratio was 1.59 (95% CI, 1.08–2.34, *p* = 0.02). In the stratified analysis, we found that older aged patients (≥65 years) were more susceptible to iNO-associated kidney injury than younger patients (*p* = 0.05).

**Conclusions:**

This study showed that iNO substantially increased the risk of renal dysfunction in patients with ARDS. Older aged patients were especially susceptible to this adverse event.

**Electronic supplementary material:**

The online version of this article (doi:10.1186/s13054-016-1566-0) contains supplementary material, which is available to authorized users.

## Background

Inhaled nitric oxide (iNO) is a rescue therapy for severe hypoxemia in patients with acute respiratory distress syndrome (ARDS). Inhaled nitric oxide gas administered via a gas mixture with the patient’s inhaled breath reaches only normally ventilated lung units and causes selective dilatation of the vessels surrounding normal alveoli [1]. This improves the ventilation-perfusion mismatch in patients with hypoxaemic respiratory failure due to acute lung injury. Although the routine use of iNO in ARDS is not recommended based on current evidence [2, 3], iNO has a substantial effect in improving oxygenation and is still frequently used in many institutions [4, 5].

Previous studies suggest that iNO has a good safety profile [6, 7]. When iNO was first introduced, common safety concerns based on pharmacological knowledge included formation of methaemogloblin, production of reactive nitrogen species, hypotension and platelet inhibition [6, 8, 9], but nephrotoxicity was not a major concern. However, a clinical trial of ARDS published in 1999 reported that iNO potentially doubled the risk of the need for renal replacement therapy (RRT) compared with controls [10]. We recently performed a systematic review and meta-analysis to evaluate the association between iNO exposure and renal dysfunction in randomized controlled trials (RCTs), and found that iNO increased the risk of acute kidney injury by 50% in patients with ARDS [11].

However, the risk estimated from our meta-analysis of RCTs may not accurately reflect the risk in daily clinical practice because RCTs often exclude patients with severe organ dysfunction and haemodynamic instability. Excluding unstable patients who are particularly vulnerable to drug-induced kidney injury in clinical trials may underestimate the risk of drug-induced nephrotoxicity [12]. Another concern about the results of meta-analysis is that study-level analyses are unable to adjust for competing risks of death. Furthermore, the information about how the baseline renal function and risk factors of drug-induced nephrotoxicity modify the risk of iNO-associated renal dysfunction is useful for clinicians but has not been evaluated.

Therefore, we conducted a cohort study to evaluate the risk of RRT associated with iNO therapy in daily clinical practice and to explore the effect modification by age, baseline renal function, shock and disease severity. We hypothesized that iNO use in ARDS is associated with an increased risk of the use of RRT. 

## Methods

### Data source and study population

**Fig. 1:**
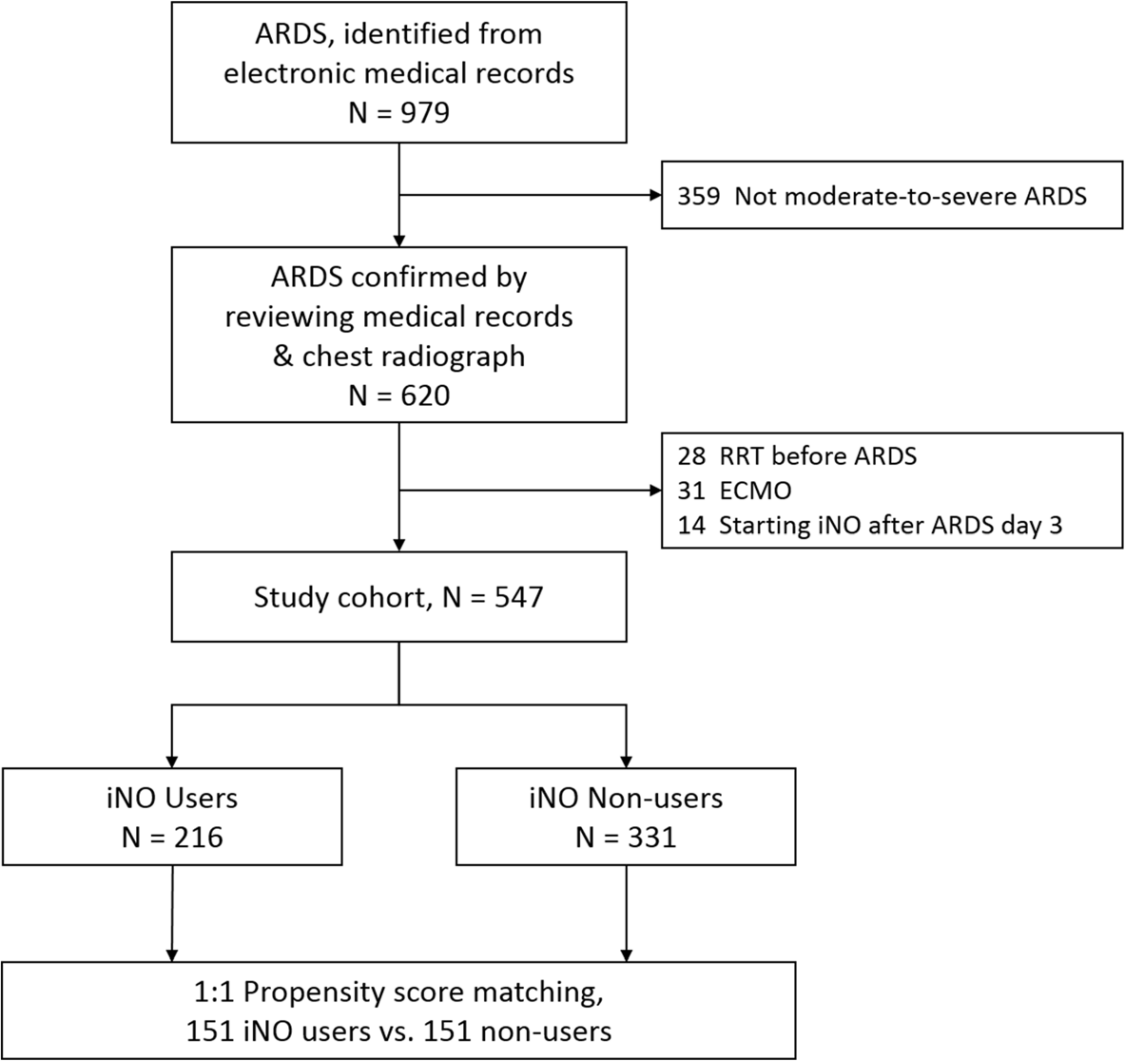
Process for selection of study subjects. *ARD*S acute respiratory distress syndrome, *iNO* inhaled nitric oxide, *ECMO* extracorporeal membrane oxygenation, *RRT* renal replacement therapy

This study was conducted using the electronic medical records and web-based imaging system of the National Taiwan University Hospital, a tertiary medical centre in northern Taiwan. We assembled a base cohort composed of all patients with a diagnosis of ARDS recorded in the admission or discharge notes of the electronic medical records between 1 January 2007 and 31 March 2015. Patients were included if they were ≥20 years of age and were managed in the ICU with invasive mechanical ventilation. We evaluated the clinical course and serial chest radiographs of each case in the base cohort to identify those patients who fulfilled the moderate or severe ARDS diagnostic criteria using the Berlin definition published in 2012 [13]. We excluded patients with mild ARDS (partial arterial oxygen pressure (PaO_2_)/fraction of inspired oxygen (FiO_2_) above 200 mmHg) to avoid diversity in the case definition before and after 2012. We also excluded patients who had received RRT before the onset of ARDS, patients who received extracorporeal life support and patients starting iNO therapy more than 3 days after the onset of ARDS. The patients who were started on iNO therapy more than 3 days after the onset of ARDS were excluded, because this study aimed to use incident user design. Incident user design has the advantages of avoiding erroneous adjusting for consequences of treatment and reducing the chances of immortal time bias [14]. Figure 1 shows the selection process of study subjects.

### Exposure and outcome

Patients who received any dose of iNO were defined as iNO users. There was no standard protocol for iNO therapy for the treatment of patients with ARDS in the study hospital. The initiation and discontinuation of iNO were at the discretion of the primary care team. The primary outcome of interest was incident RRT including intermittent haemodialysis, sustained low efficiency dialysis and continuous RRT. All patients were followed from the onset of ARDS until the initiation of RRT, or until censored on death from any cause, transfer to another hospital or 30-day follow up after the onset of ARDS, whichever occurred first.

### Covariates

We obtained patient data from the medical records including age, sex, year of cohort entry, body height and weight, aetiology of ARDS, Simplified Acute Physiology Score II (SAPS II) [15], Lung Injury Score [16], comorbidities, ventilator settings, vital signs, arterial blood gas, complete blood cell count, creatinine and bilirubin levels, urine output, radiographic pattern and vasopressor use on the first day of ARDS. Creatinine clearance was estimated using the Cockcroft-Gault formula [17]. In the study hospital, patients with ARDS were ventilated using tidal volumes and PEEP settings recommended by the ARDS network [18].

### Statistical analysis

Cox proportional hazard modelling was used to calculate the crude hazard ratio and propensity-adjusted cause-specific hazard ratio for RRT. We computed the propensity score using logistic regression with receipt of iNO therapy as the dependent variable. Propensity-based matching was used to select control patients who were similar to patients receiving treatment, matching on many confounders simultaneously [19]. For matching, a caliper width of 0.2 times the standard deviation of the propensity score without replacement was used [20]. Using the propensity-matched cohort, we analysed data by intention-to-treat analysis and calculated the point estimates and 95% confidence intervals (95% CI) of the treatment effect.

Because the important assumption of non-informative censoring required for a Kaplan-Meier estimator and Cox proportional hazard model might be violated in our case due to the presence of a competing risk of death [21], we used a cumulative incidence function rather than a Kaplan-Meier estimator to estimate the probabilities of incident RRT over time. Our primary analysis used the model proposed by Fine and Gray to account for competing risks due to death [22].

In addition, we assessed the strength of an unmeasured confounder needed to move the observed effect to the null using the rule-out approach proposed by Schneeweiss [23], because there may be unmeasured confounders inherent to the nature of the design of this observational study. To explore possible effect modification by age, baseline renal function, shock and disease severity based on biological plausibility [24, 25], we used stratified analysis to estimate the risk in each subgroup. All analyses were conducted with Stata version 11 (StataCorp, TX, USA).

## Results

A total of 547 patients met the study inclusion criteria, including 216 iNO users and 331 non-users. The process for selection of study subjects is provided in Fig. 1. The mean patient age was 63 ± 17 years. At study entry, 313 patients (57.2%) had moderate ARDS and 234 (42.8%) had severe ARDS. The major causes of ARDS were pneumonia (77.7%) and non-pulmonary sepsis (14.4%). The median exposure duration of iNO for iNO users was 4 days (interquartile range, 2–7 days) and median initial dose of iNO was 20 ppm (interquartile range, 15-20). The iNO users had worse oxygenation, higher Lung Injury Scores, higher plateau pressure and higher mean airway pressure compared with the non-users (Table 1). After one-to-one matching for pretreatment covariates, 151 iNO users were matched with iNO non-users. Table 1 presents the baseline characteristics of the patients in the overall and propensity-matched cohorts. The propensity score distributions among iNO users and non-users and the 30 variables used in the propensity score model are provided in Additional file 1 (Additional file 1: Table S1 and Figure S1). The overall 30-day mortality for the total cohort and propensity-matched cohort was 42% and 43% respectively.

**Table 1:** Baseline characteristics of patients before and after propensity matching

Characteristic	Overall cohort (n = 547)			Propensity-matched cohort (n = 302)		
	iNO users (n = 216)	Non-users (n = 331)	*P* value	iNO users (n = 151)	Non-users (n = 151)	*P* value
Age, years	61 ± 17	64 ± 16	0.07	63 ± 17	62 ± 16	0.59
Female sex, *n* (%)	66 (30.6)	114 (34.4)	0.34	48 (31.8)	50 (33.1)	0.81
ARDS severity						
Moderate, *n* (%)	105 (49)	208 (63)	0.001	82 (54)	74 (49)	0.36
Severe, *n* (%)	111 (51)	123 (37)		69 (46)	77 (51)	
Body-mass index, kg/m^2^	23.3 ± 4.7	21.9 ± 3.9	<0.001	22.7 ± 4.7	22.7 ± 4.0	0.95
Cause of ARDS, *n* (%)						
Pneumonia	167 (77.3)	258 (78.0)	0.52	121 (80.1)	122 (80.8)	0.93
Non-pulmonary sepsis	32 (14.8)	47 (14.2)		20 (13.3)	19 (12.6)	
Acute interstitial pneumonia	11 (5.1)	10 (3.0)		7 (4.6)	5 (3.3)	
Multiple transfusion	1 (0.5)	5 (1.5)		1 (0.7)	2 (1.3)	
Others	5 (2.3)	11 (3.3)		2 (1.3)	3 (2.0)	
Renal function						
Creatinine, mg/dL	1.1 (0.8–1.6)	1.1 (0.8 − 1.8)	0.75	1.1 (0.8 − 1.6)	1.0 (0.8 − 1.7)	0.83
Creatinine clearance, mL/min	57.0 (35.5 − 82.2)	48.3 (31.0 − 70.2)	0.01	55.2 (35 − 81)	53.8 (33.3–81.5)	0.97
AKI stage 1, *n* (%)	57 (78)	91 (79)		39 (81)	42 (84)	
AKI stage 2, *n* (%)	9 (12)	13 (11)	0.98	5 (10)	4 (8)	0.91
AKI stage 3, *n* (%)	7 (10)	11 (10)		4 (8)	4 (8)	
Shock, *n* (%)	91 (42.1)	117 (35.4)	0.11	56 (37.1)	56 (37.1)	1.00
SAPS II	49.9 ± 15.1	49.8 ± 14.7	0.95	49.9 ± 14.2	50.2 ± 15.2	0.89
Lung injury score, total score	11.4 ± 1.9	10.95 ± 1.9	0.005	11.3 ± 1.8	11.5 ± 1.8	0.46
FiO_2_	0.9 (0.6–0.9)	0.7 (0.6–1.0)	<0.001	0.8 (0.6–1.0)	0.8 (0.6–1.0)	0.96
PaO_2_/FiO_2_	96 (68 − 134)	123 (84–181)	<0.001	104 (74–143)	99 (66 − 140)	0.45
PEEP, cm H_2_O	8.9 ± 3.6	8.1 ± 3.0	0.002	8.3 ± 3.4	8.7 ± 3.2	0.35
Tidal volume, mL/pBW	8.7 ± 1.9	8.5 ± 2.0	0.23	8.7 ± 1.9	8.6 ± 2.0	0.82
pH	7.38 ± 0.10	7.39 ± 0.09	0.35	7.39 ± 0.10	7.40 ± 0.08	0.66
PaO_2_, mmHg	82.0 ± 33.2	94.2 ± 43.4	<0.001	83.9 ± 35.0	80.9 ± 32.0	0.45
PaCO_2_,mmHg	37.5 ± 10.4	37.1 ± 10.5	0.67	37.4 ± 9.9	37.3 ± 8.7	0.97
HCO_3_^-^, mmol/L	22.0 ± 5.1	22.5 ± 5.6	0.27	22.3 ± 5.2	22.5 ± 4.7	0.66
Static respiratory compliance, mL/cm-H_2_O	29.6 ± 12.8	29.7 ± 13.1	0.92	29.0 ± 12.4	30.2 ± 13.7	0.45
Plateau pressure, cm H_2_O	28 ± 6.8	26 ± 7.3	0.03	27 ± 6.1	27 ± 7.9	0.70
Driving pressure (cm H_2_O)	18.8 ± 6.4	18.2 ± 6.6	0.35	18.5 ± 5.6	18.5 ± 7.1	0.95
Mean airway pressure, cm H_2_O	15.7 ± 4.3	14.5 ± 3.9	0.002	15.0 ± 4.1	15.2 ± 3.8	0.62
Duration of mechanical ventilation, days	11 (6–20)	12 (7–24)	0.09	12 (6–22)	12 (6–21)	0.93

Data are expressed as the mean ± standard deviation or median (interquartile range) unless otherwise specified. Detailed variables used in the propensity score model are listed in Additional file 1: Table S1. Abbreviations: *ARDS* acute respiratory distress syndrome, *AKI* acute kidney injury, *iNO* inhaled nitric oxide, *PEEP* positive end-expiratory pressure, *pBW* predicted body weight, *SAPS* Simplified Acute Physiology Score

The crude risk for the need for RRT was significantly higher in iNO users compared with non-users (37% vs. 21%, *p* < 0.001), although the baseline estimated creatinine clearance was better in iNO users (60.9 vs. 54.5 mL/minute, *p* = 0.04, Table 1). The crude hazard ratio was 2.23 (95% CI 1.61–3.09, *p* < 0.001).

**Fig. 2:**
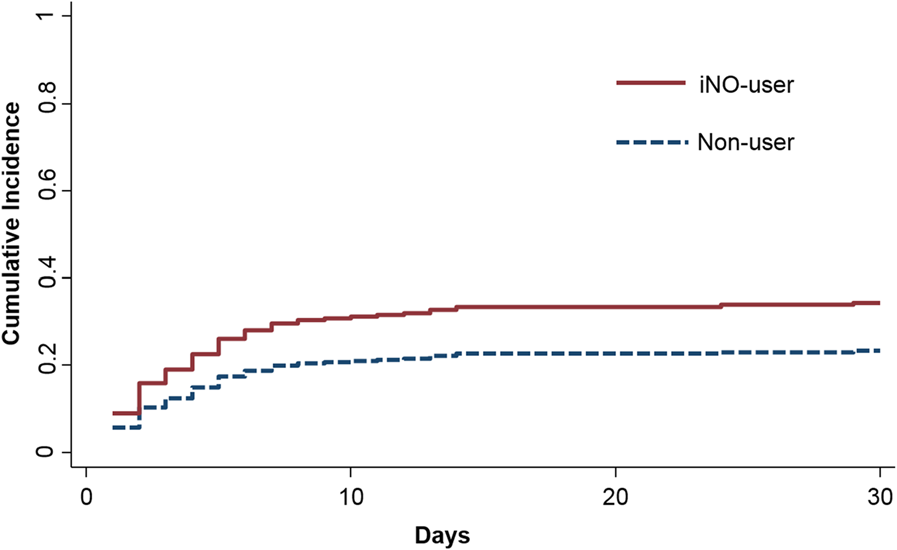
The cumulative incidence of the initiation of renal replacement therapy for inhaled nitric oxide *(iNO*) users and non-users in the propensity-matched cohort

Figure 2 depicts the cumulative incidence of RRT over time using the propensity-matched cohort. The 30-day cumulative incidence of RRT was 34% and 23% for iNO users and non-users, respectively. Table 2 summarizes the adjusted hazard ratios of incident RRT estimated by different models. The primary analysis with Fine-Gray competing-risks regression using the propensity-matched cohort showed that the adjusted hazard ratio of iNO users compared with non-users was 1.59 (95% CI 1.08–2.34, *p* = 0.02). The cause-specific hazard ratio from the Cox proportional hazard model, treating death as censoring, was 1.76 (95% CI 1.19–2.60, *p* = 0.005).

**Table 2:** Primary analysis of the hazard ratio of renal replacement therapy associated with inhaled nitric oxide (iNO)

Model	Events/person days, number		Hazard ratio (95% CI)	*P* value
	iNO	Control		
Crude hazard ratio	79/2175	69/5417	2.23 (1.61 to 3.09)	<0.001
Multiple regression Cox model^a^	79/2175	69/5417	2.17 (1.48 to 3.20)	<0.001
Cause-specific Cox model, propensity-matched cohort	52/1600	36/2370	1.76 (1.19 to 2.60)	0.005
Fine-Gray competing-risks regression, propensity-matched cohort	52/1600	36/2370	1.59 (1.08 to 2.34)	0.02

^a^Adjusted for all variables used in the logistic regression model for the propensity score

**Fig. 3:**
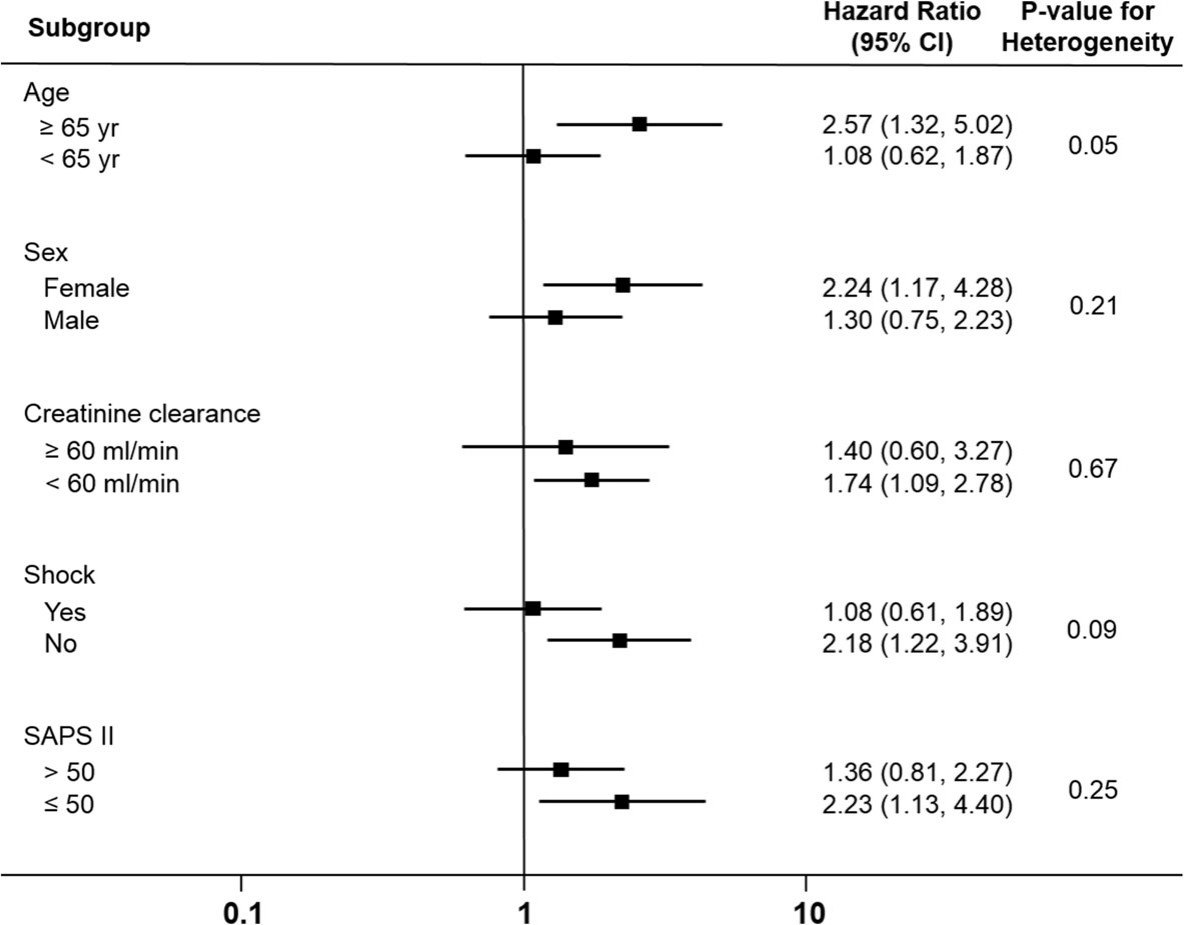
Adjusted hazard ratios for the need for renal replacement therapy in patients treated with inhaled nitric oxide compared with that of non-users in the pre-specified subgroups. *SAPS* Simplified Acute Physiology Score

Figure 3 shows the results of the pre-specified subgroup analyses based on competing-risk regression using the propensity-matched cohort. In the subgroup analysis, older age (age ≥65 years) was associated with a higher risk of iNO-associated RRT compared with younger age (*p* value for interaction 0.05). The risk of iNO-associated RRT was also higher among female patients and patients with lower creatinine clearance, absence of shock and lower SAPS II scores compared with their counterparts, but these findings were not statistically significant on interaction.

Additional file 1: Figure S2 shows the results of sensitivity analysis to determine whether an unmeasured binary confounder could explain a hazard ratio of this magnitude based on competing risk regression using the propensity-matched cohort. The x-axis represents the hypothetical hazard ratio for RRT associated with the unmeasured confounder and the y-axis represents the hypothetical association between the confounder and iNO use.

## Discussion

This is the first study to evaluate the risk of iNO-associated renal dysfunction in daily clinical practice. We found that among patients with ARDS, exposure to iNO significantly increased the risk of need for RRT, and that patients ≥65 years old were especially prone to this hazard. Our results are consistent with the data from prior RCTs, which have shown that iNO therapy increases the risk for renal dysfunction in patients with ARDS by 50% [11, 26]. Even though we used competing-risk analysis for a conservative estimation of the event risk, the risk of RRT increased by 59% in patients receiving iNO therapy in this cohort study. Based on the results of this study and the pooled data from randomized trials [11, 26], we suggest that clinicians monitor renal function in patients receiving iNO and exercise caution with the concurrent use of nephrotoxic agents and iNO therapy.

A number of risk factors enhance the vulnerability of the kidney to the nephrotoxic effects of drugs and are usually categorized as patient-specific, kidney-related, and drug-related factors [25]. Old age, female sex and chronic kidney disease are important patient-specific factors associated with increased vulnerability to drug-related kidney injury [12, 25]. In the subgroup analysis of our study (Fig. 3), older patients were more prone to iNO-associated renal dysfunction. We also observed a higher risk of iNO-associated renal dysfunction in female patients and in patients with impaired baseline renal function, but these factors were not found to be significant effect modifiers . Shock is also an important risk factor for acute kidney injury among critically ill patients [27]. In the subgroup analysis, we did not observe a synergistic effect of iNO and shock on the risk of renal dysfunction. In contrast, the effect of iNO on renal dysfunction appeared to be obscured by the presence of shock. We speculate that this might be related to the dominant effect of shock on the development of acute kidney injury via the mechanism of ischaemic kidney injury [28].

Emerging data have revealed the phenomenon and mechanism of lung-kidney cross-talk [29]. Positive-pressure ventilation and PEEP alters venous return, neurohormonal system, pulmonary vascular resistance, and right ventricular function. High PEEP is associated with renal dysfunction [30], and right ventricular dysfunction may contribute to alterations of renal perfusion and oxygenation [29]. The prevalence of acute cor pulmonale in ARDS is estimated to be 20–30% [31]. If iNO was used to treat cor pulmonale or its use was associated with a higher airway pressure setting, a biased association between iNO exposure and renal dysfunction may be observed in the context of lung-kidney cross-talk. In this study, pulmonary arterial pressure was seldom measured and iNO therapy was usually initiated for hypoxaemia. PEEP levels, mean airway pressure and plateau pressure are similar in iNO users and non-users. Lung-kidney cross-talk cannot explain the excessive risk of renal dysfunction observed in the iNO-treated group. It is noteworthy that the PEEP levels in this study were lower than the levels recommended in the literature. This situation is not uncommon in observational studies because there are barriers to achieving the suggested PEEP levels in clinical practice. In the LUNG SAFE study [4], a large epidemiology study of ARDS management in real-world practice, the observed PEEP levels (8.4 cm H_2_O) were similar to the PEEP levels in our study.

The mechanisms accounting for iNO-associated renal dysfunction are unclear. The metabolites and by-products of nitric oxide may play a role. Nitric oxide is metabolized in the blood in several ways [32]. First, iNO interacts with dissolved O_2_ to form NO_2_^-^. Also, iNO interacts with oxyhaemoglobin to form methaemoglobin, which is in turn reduced back to haemoglobin and NO_3_^-^ [33]. Finally, nitric oxide can combine with deoxyhaemoglobin to form nitrosohaemoglobin, or it may combine with carrier molecules to form S-nitrosothiols [7]. These nitric oxide metabolites can increase protein nitrosation and increase the oxidative load [34]. With respect to the renal effects of these metabolites and by-products of iNO, excessive methaemoglobin may result in tissue hypoxia; however, the formation of methaemoglobin is usually insignificant in iNO doses below 20 ppm [6]. In a previous animal study, systemically circulating NO_2_^-^ may have led to cytotoxic effects on renal parenchymal cells [35]. Future studies are needed to determine the pathways of iNO associated renal dysfunction.

We performed sensitivity analysis to address whether an unmeasured binary confounder could explain the hazard ratio of the observed effect. Additional file 1: Figure S2 shows the results of our sensitivity analysis. For example, we supposed that the use of a nephrotoxic drug was a potential unmeasured confounder and was used in 40% of the patients (green line). If the drug was used fourfold more frequently in iNO-users than in non-users (y-axis), and the risk of renal dysfunction increased more than fourfold (x-axis), then the drug alone could itself account for the observed association between iNO exposure and renal dysfunction. However, such a scenario is very unlikely in actual clinical practice because no known commonly used interventions in ARDS are associated with high nephrotoxicity (increased fourfold risk of initiating RRT). For the potential confounders with prevalence of 10% (blue line), these confounders must be associated with more than fivefold increased risk of renal dysfunction. These results suggest that the association that we observed between iNO exposure and renal dysfunction is unlikely to be due solely to confounding.

Using RRT as a surrogate measure of nephrotoxic effects may introduce bias if RRT was initiated for indications other than iNO-associated renal failure or if the physicians considered different thresholds for initiation of RRT for iNO users and non-users. Bias related to the former aspect would be limited because the factors associated with initiation of RRT, such as comorbidity, shock and baseline renal function, were well-balanced in the two groups after propensity-score matching. In addition, this bias would not alter the study conclusion because non-differential misclassification leads the true risk to the null value.

Our study has several limitations. First, this is a single-centre study, and the risk of iNO-associated renal dysfunction observed in this study may not be generalizable to patients in other hospitals. Second, there was no standard protocol for iNO therapy for the treatment of patients with ARDS in the National Taiwan University Hospital. The initiation and discontinuation of iNO were at the discretion of the primary care team. It is unclear whether the preference for iNO therapy affected the practice on RRT. We used propensity score matching in this study to balance the baseline covariates among iNO users and non-users to decrease confounding by indication. Finally, there may be unmeasured confounders inherent in the design of this observational study. We performed sensitivity analysis (Additional file 1: Figure S2) to address whether an unmeasured binary confounder could explain the hazard ratio of the observed effect. The results of the sensitivity analysis suggest that the observed association between iNO and renal dysfunction is unlikely to be solely due to confounding.

## Conclusions

This study used data from a clinical practice environment to evaluate the risk of iNO-associated renal dysfunction in terms of newly initiated RRT. We found that iNO was associated with an increased risk of the need for RRT in patients with ARDS, especially in patients ≥65 years old. Clinicians should monitor renal function and avoid the concurrent use of nephrotoxic agents during iNO therapy. In addition, we advise the monitoring and reporting of renal function in future iNO trials.

## Additional file

Additional file 1: Table S1. Baseline variables used for propensity matching. **Figure S1.** Propensity score distribution for the users and non-users of inhaled nitric oxide (*iNO*). **Figure S2.** The strength of an unmeasured confounder needed to move the observed effect to the null. (PDF 410 kb)

## Abbreviations

AKI: acute kidney injury
ARDS: acute respiratory distress syndrome
FiO_2_: fraction of inspired oxygen
iNO: inhaled nitric oxide
PaO_2_: partial arterial oxygen pressure
PEEP: positive end expiratory pressure
RCT: randomized controlled trial
RRT: renal replacement therapy
SAPS: Simplified Acute Physiology Score
