# Supplementary material for: Inhaled nitric oxide and the risk of renal dysfunction in patients with acute respiratory distress syndrome: a propensity-matched cohort study
Source: Crit Care. 2016 Nov 30;20:389. doi: 10.1186/s13054-016-1566-0 (PMC5131425; doi:10.1186/s13054-016-1566-0)

Additional electronic files

Inhaled nitric oxide and the risk of renal dysfunction in patients with acute respiratory distress syndrome: a propensity-matched cohort study

Contents **Page**

Table S1. Baseline variables used for propensity matching ………………………… 2

Fig. S1. Propensity score distribution for the users and non-users of inhaled nitric oxide (iNO) ………………………………………………………… 6

Fig. S2. The strength of an unmeasured confounder needed to move the observed effect to the null ………………………………………………………. 7

Table S1. Baseline Variables Used for Propensity Matching

Characteristic	Overall cohort (N= 547)			Propensity-matched cohort (N= 312)		
	iNO users (N=216)	Non-users (N=331)	p-value	iNO users (N=151)	Non-users (N=151)	p-value
Age (yr)	61 ± 17	64 ± 16	0.07	63 ± 17	62 ± 16	0.59
Female, n (%)	66 (30.6)	114 (34.4)	0.34	48 (31.8)	50 (33.1)	0.81
Medical ICU, n (%)	187 (86.6)	248 (74.9)	0.001	128 (84.8)	132 (87.4)	0.51
Height (cm)	162.8 ± 8.7	161.8 ± 8.1	0.16	162.5 ± 8.3	162.6 ± 8.4	0.94
Weight (kg)	62 (51-70.5)	57 (49-64)	<0.001	60 (50-69)	60 (50-68)	0.86
Community-acquired ARDS, n (%)	163 (75.4)	251 (75.8)	0.92	111 (73.5)	116 (76.8)	0.44
Cause of ARDS						
Pneumonia	167 (77.3)	258 (78.0)	0.52	121 (80.1)	122 (80.8)	0.93
Non-pulmonary sepsis	32 (14.8)	47 (14.2)		20 (13.3)	19 (12.6)	
Acute interstitial pneumonia	11 (5.1)	10 (3.0)		7 (4.6)	5 (3.3)	
Multiple transfusion	1 (0.5)	5 (1.5)		1 (0.7)	2 (1.3)	
Others	5 (2.3)	11 (3.3)		2 (1.3)	3 (2.0)	
Study year						
Year 1, n (%)	37 (17.1)	27 (8.2)	0.001	19 (12.6)	21 (13.9)	0.97
Year 2, n (%)	37 (17.1)	55 (16.6)		28 (18.5)	27 (17.9)	
Year 3, n (%)	47 (21.8)	59 (17.8)		35 (23.2)	33 (21.9)	
Year 4, n (%)	38 (17.6)	49 (14.8)		23 (15.2)	25 (16.6)	

Year 5, n (%)	35 (16.2)	83 (25.1)		28 (18.5)	31 (20.5)	
Year 6, n (%)	15 (6.9)	26 (7.9)		11 (7.3)	10 (6.6)	
Year 7, n (%)	7 (3.2)	32 (9.7)		7 (4.6)	4 (2.7)	
Renal function profiles						
Baseline Creatinine clearance (mL/min)	63.4 (40.6-89.5)	54 (34.8-76.9)	0.01	56.8 (39.2-85.0)	60.5 (37.9-86.9)	0.66
Creatinine clearance on ARDS day 1 (mL/min)	57.0 (35.5-82.2)	48.3 (31.0-70.2)	0.01	55.2 (35-81)	53.8 (33.3-81.5)	0.97
Urine output (mL/kg)	1.00 ± 0.77	1.03 ± 0.83	0.66	1.01 ± 0.71	1.04 ± 0.83	0.76
Acute kidney injury, n (%)	73 (33.8)	115 (34.7)	0.82	48 (31.8)	50 (33.1)	0.81
Stage 1, n (%)	57 (78)	91 (79)	0.98	39 (81)	42 (84)	0.91
Stage 2, n (%)	9 (12)	13 (11)		5 (10)	4 (8)	
Stage 3, n (%)	7 (10)	11 (10)		4 (8)	4 (8)	
Body temperature (°C)						
<35.5	4 (1.9)	11 (3.3)	0.19	4 (2.7)	3 (2.0)	0.99
35.5≤ to <36	8 (3.7)	18 (5.4)		4 (2.7)	3 (2.0)	
36≤ to <38	120 (55.6)	152 (45.9)		79 (52.3)	79 (52.3)	
38≤ to <39	61 (28.2)	102 (30.8)		46 (30.5)	47 (31.1)	
39 ≤	23 (10.7)	48 (14.5)		18 (11.9)	19 (12.6)	
Heart rate (f/min)						

<70	4 (1.9)	10 (3.1)	0.93	4 (2.7)	4 (2.7)	0.99
70≤ to <80	9 (4.2)	12 (3.7)		6 (4.0)	5 (3.3)	
80≤ to <100	48 (22.5)	74 (22.8)		31 (20.5)	35 (23.2)	
100≤ to <120	65 (30.5)	104 (32.0)		49 (32.5)	48 (31.8)	
120≤ to <140	56 (26.3)	76 (23.4)		41 (27.2)	38 (25.2)	
140≤	31 (14.6)	49 (15.1)		20 (13.3)	21 (13.9)	
White blood cell count (/µL)						
<2000	20 (9.7)	21 (6.6)	0.53	12 (8.0)	14 (9.3)	0.94
2000≤ to <4000	11 (5.3)	20 (6.3)		10(6.6)	8 (5.3)	
4000≤ to <9000	69 (33.5)	96 (30.0)		50 (33.1)	49 (32.5)	
9000≤ to <20000	85 (41.3)	150 (46.9)		61 (40.4)	65 (43.1)	
20000≤	21 (10.2)	33 (10.3)		18 (11.9)	15 (9.9)	
Opacities on chest radiograph						
2 quadrants, n (%)	52 (24.1)	65 (19.6)	0.46	30 (19.9)	34 (22.5)	0.79
3 quadrants, n (%)	58 (26.9)	92 (27.8)		40 (26.5)	36 (23.8)	
4 quadrants, n (%)	106 (49.1)	174 (52.6)		81 (53.6)	81 (53.6)	
Bacteremia or fungemia, n (%)	50 (23.2)	52 (15.7)	0.03	26 (17.2)	29 (19.2)	0.66
Shock, n (%)	91 (42.1)	117 (35.4)	0.11	56 (37.1)	56 (37.1)	1.00
SAPS II	49.9 ± 15.1	49.8 ± 14.7	0.95	49.9 ± 14.2	50.2 ± 15.2	0.89
Lung injury score (Total score)	11.4 ± 1.9	10.95 ± 1.9	0.005	11.3 ± 1.8	11.5 ± 1.8	0.46
PaO2/FiO2, mmHg	96 (68-134)	123 (84-181)	<0.001	104 (74-143)	99 (66-140)	0.45
$PaCO_2$ (mmHg)	37.5 ± 10.4	37.1 ± 10.5	0.67	37.4 ± 9.9	37.3 ± 8.7	0.97

Comorbidity before ARDS						
Stroke, n (%)	17 (7.9)	27 (8.2)	0.90	12 (8.0)	14 (9.3)	0.68
Coronary artery disease, n (%)	15 (6.9)	30 (9.1)	0.38	12 (8.0)	12 (8.0)	1.00
Heart failure, n (%)	15 (6.9)	23 (7.0)	1.00	8 (5.3)	8 (5.3)	1.00
Cholestatic liver disease, n (%)	21 (9.7)	24 (7.3)	0.30	12 (8.0)	11 (7.3)	0.83
Obstructive airway diseases, n (%)	18 (8.3)	40 (12.1)	0.16	16 (10.6)	18 (11.9)	0.72
Structure lung diseases, n (%)	27 (12.5)	33 (10.0)	0.36	19 (12.6)	20 (13.3)	0.86
Solid cancer, n (%)	57 (26.4)	113 (34.1)	0.06	46 (30.5)	50 (33.1)	0.62
Hematological cancer, n (%)	34 (15.7)	40 (12.1)	0.22	20 (13.3)	20 (13.3)	1.00

Data are measured on the ARDS day 1 and expressed as the mean ± standard deviation or median (interquartile range) unless otherwise specified.

ARDS, Adult respiratory distress syndrome

SAPS, Simplified Acute Physiology Score

PEEP, Positive end-expiratory pressure

Fig. S1. Propensity score distribution for the users and non-users of inhaled nitric oxide (iNO).

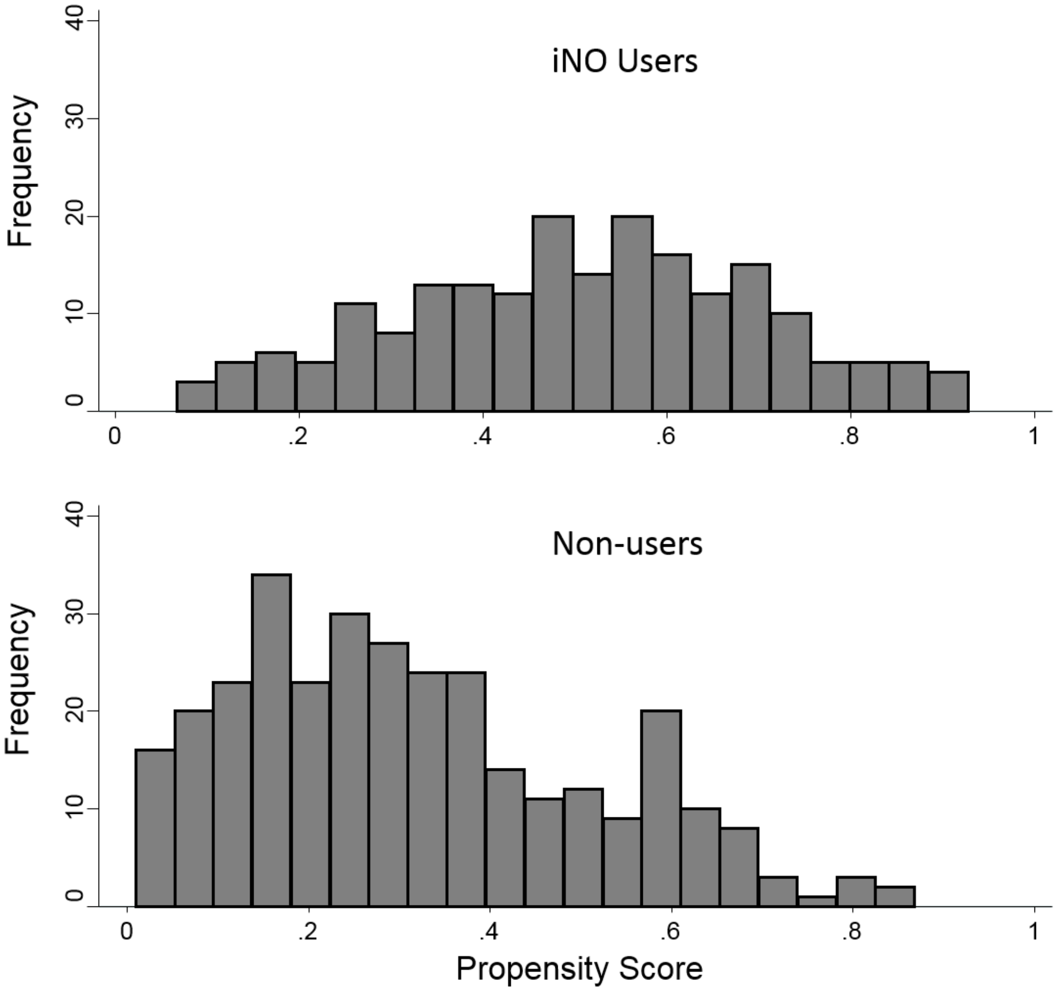

Fig. S2. The strength of an unmeasured confounder needed to move the observed effect to the null. iNO, inhaled nitric oxide. RRT, renal replacement therapy.

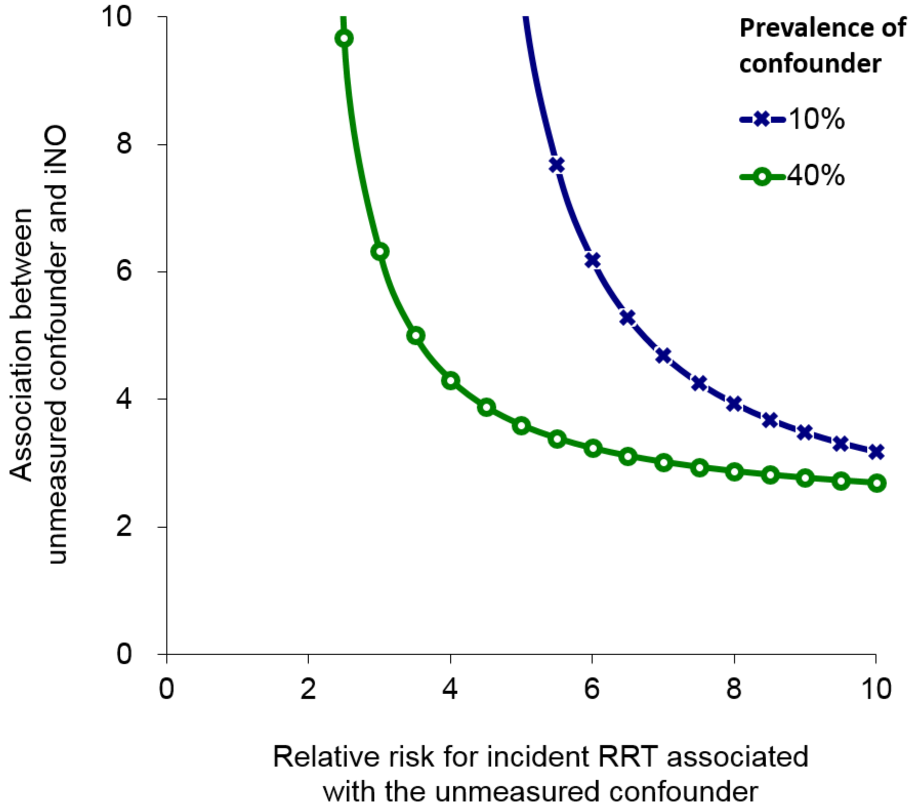

Supplement: Additional file 1: Table S1. — Baseline variables used for propensity matching. Figure S1. Propensity score distribution for the users and non-users of inhaled nitric oxide (iNO). Figure S2. The strength of an unmeasured confounder needed to move the observed effect to the null. (PDF 410 kb) [file 13054_2016_1566_MOESM1_ESM.pdf]
